# Cell-Free Biological Approach for Corneal Stromal Wound Healing

**DOI:** 10.3389/fphar.2021.671405

**Published:** 2021-05-28

**Authors:** Vishal Jhanji, Isabelle Billig, Gary Hin-Fai Yam

**Affiliations:** Department of Ophthalmology, University of Pittsburgh, Pittsburgh, PA, United States

**Keywords:** cornea, wound healing, fibrosis, protein therapy, microRNAs

## Abstract

Corneal opacification is the fourth most common cause of blindness globally behind cataracts, glaucoma, and age-related macular degeneration. The standard treatment of serious corneal scarring is corneal transplantation. Though it is effective for restoring vision, the treatment outcome is not optimal, due to limitations such as long-term graft survival, lifelong use of immunosuppressants, and a loss of corneal strength. Regulation of corneal stromal wound healing, along with inhibition or downregulation of corneal scarring is a promising approach to prevent corneal opacification. Pharmacological approaches have been suggested, however these are fraught with side effects. Tissue healing is an intricate process that involves cell death, proliferation, differentiation, and remodeling of the extracellular matrix. Current research on stromal wound healing is focused on corneal characteristics such as the immune response, angiogenesis, and cell signaling. Indeed, promising new technologies with the potential to modulate wound healing are under development. In this review, we provide an overview of cell-free strategies and some approaches under development that have the potential to control stromal fibrosis and scarring, especially in the context of early intervention.

## Introduction

The cornea is the clear window of the eyes. The rays of light pass through the cornea before reaching the retina. Its main functions include 1) mechanical protection of inner eye tissues from physical, chemical, and UV damages, 2) an optically clear structure for the undisturbed passage of light, 3) a highly refractive medium (providing two-thirds of the eye’s refractive power) to converge the incident light rays through the pupil and focus on the fovea, and 4) an immune barrier (formed by the corneal epithelium and tear film) from external agents. Anatomically, the cornea has three cellular layers (epithelium, stroma, and endothelium) in a sandwich with two acellular layers: the Bowman’s layer and Descemet’s membrane. The corneal stroma forms about 90% of the overall corneal thickness, giving the strength and structural integrity of the cornea. The cornea’s transparency is maintained by the regular alignment of collagen fibers and the relative dehydration status inside the stroma. Besides, corneal transparency is associated with corneal avascularity and the self-renewal of corneal epithelium.

Corneal transparency is critical for optimal vision. Damage to the cornea by trauma or infection can cause irreversible reduction of transparency, leading to distorted vision and eventually blindness. Corneal blindness is a leading cause of vision loss, with 4.2 million people worldwide affected by corneal opacities (World Report on Vision, World Health Organization, 2019). Affected individuals lose their independence and have a poor quality of life. Treatment for corneal injury is determined by several factors that include etiology, location, and severity of the injury. First-line treatments for corneal abrasions are antibiotics and lubricants, while mild to moderate pain can be controlled with nonsteroidal anti-inflammatory drugs. When corneal pathologies cause severe and irreversible scarring, a corneal transplant often is the best solution. In a global survey, more than 180,000 cases of penetrating keratoplasty (PK) were conducted in 116 countries and 284,000 corneas were procured in 2012 (Gain et al., 2016). An Australian study of 18,686 penetrating corneal grafts reported that more than 70% of patients with multiple indications showed visual improvement in short-term outcome, however, universal attrition of visual acuity was observed in a follow-up study of patients up to 22°years post-transplant (Williams et al., 2008). Reasons for treatment failures include irreversible graft rejection (34%), corneal endothelial failure or glaucoma (24%), and infection (14%). By 2°years post-surgery, only 45% of grafted eyes showed the best-corrected visual acuity (BCVA) of 6/12 or better and 26% had BCVA less than 6/60. Even more problematic, the global shortage of transplantable donor corneal tissues has greatly hindered the availability of corneal transplantation. In fact, as of now, only one cornea is available for every 70 patients worldwide (Gain et al., 2016).

After more than a century of full-thickness corneal transplantation on patients (the first surgery by Dr. Eduard Zirm in 1905 (Zirm, 1906)), the clinical practice has been tremendously improved with advances in microsurgical techniques and instrumentation, and the use of surgical lasers (excimer and femtosecond lasers). This has resulted in different forms of selective lamellar keratoplasty (removal of the diseased corneal layer only) (Jhanji et al., 2012; Chamberlain, 2019). Specifically, anterior lamellar keratoplasty (ALK) replaces the epithelium and anterior stroma, while retaining the healthy posterior stroma and endothelium. Deep anterior lamellar keratoplasty (DALK) is performed when the stroma has to be replaced but the healthy recipient endothelium is maintained (Luengo-Gimeno et al., 2011; Arundhati et al., 2020). Compared with PK, ALK and DALK achieve better clinical outcomes, since they preserve the host corneal endothelium, thereby avoiding graft rejection due to endothelial failure. Lamellar techniques can also rescue failed penetrating grafts over successive PK, hence reducing the intraoperative risks and promoting visual recovery (Hos et al., 2019; Alio Del Barrio et al., 2021). Nonetheless, these approaches require transplantable corneal tissues, and there is an unmet need for donor corneas worldwide.

Along with recent advances in tissue engineering and scaffold biology, the discovery of corneal stromal stem cells (CSSC) presents a potentially new approach to treat corneal stromal disorders (Du et al., 2009; Basu et al., 2014; Shojaati et al., 2018). An interventional trial of CSSC to improve corneal clarity for patients with scarred corneas is underway in India (https://clinicaltrials.gov/ct2/show/NCT02948023). Another clinical trial showed that the stromal implantation of autologous adipose-derived mesenchymal stem cells improved the pachymetric and visual parameters in patients with advanced keratoconus (El Zarif et al., 2021). Different reviews have described the development of these cell-based therapeutic modalities and discussed their potential to substitute keratoplasty for corneal scarring (Fuest et al., 2016; Alio Del Barrio and Alio, 2018).

In this review, we provide an overview of conventional cell-free medications and new strategies that can be developed to control corneal fibrosis and scarring. Some of these could be applied as an early intervention to stimulate native tissue regeneration or work in combination with other treatments to enhance vision recovery.

## Corneal Stroma and Response to Injury

The corneal stroma is composed of specialized extracellular matrix (ECM) components and collagen fibrils. These fibrils are mainly type I and V collagens and are organized in parallel bundles that are aligned into layers, or lamellae. About 300 to 500 collagen lamellae are positioned in perpendicular orientations to comprise the stroma, establishing the mechanical strength and optical properties of the cornea (Meek and Knupp, 2015). Keratocytes, the dominant cell type inside the corneal stroma, are quiescent and locate between collagen lamellae (Yam et al., 2020). They produce collagens and synthesize keratan sulfate proteoglycans (KSPG; lumican, keratocan, and mimecan) that regulate collagen fibril alignment and spacing, hence keratocyte activities are crucial for the stromal organization and transparency. When the corneal stromal injury involves both epithelial and stromal elements, the release of inflammatory cytokines (mainly interleukin-1, IL-1) from the damaged epithelium induces keratocytes in the anterior stroma to undergo apoptosis (Wilson et al., 2007). Surviving keratocytes are activated and transform into proliferative fibroblastic cells, the stromal fibroblasts (SF), with repair phenotypes that initiate wound healing responses (Fini, 1999). Stimulated by serum factors, chemokines, and cytokines (e.g. transforming growth factor-beta, TGF-β; basic fibroblast growth factor, and platelet-derived growth factor, PDGF), the cells mediate Smad and non-Smad-dependent pathways, such as phosphoinositide-3 kinase (PI3K)-independent Akt and JNK signaling. SF express fibronectin receptors, produce and deposit repair-type ECM proteins (e.g. fibronectin and SPARC), and collagenases to promote tissue remodeling (Carlson et al., 2003; Chen et al., 2009; Yam et al., 2020). SF lose keratocyte phenotypes and become migratory, with the cytoskeletal arrangement and focal adhesion changes regulated by PI3K/Rac1/Rho pathways (Sloniecka et al., 2016). The presence of pro-inflammatory and chemotactic factors, such as IL-8, attracts neutrophil infiltration, further stimulating SF generation and repopulation via the P-selectin pathway (Lam et al., 2015). Studies have shown that SF express G-protein-coupled seven-transmembrane span receptor CXCR4 and respond to its ligand α-chemokine stromal-derived factor-1 (SDF-1), possibly mediating the recruitment of dendritic cells into injured corneas during inflammation, and also promote angiogenesis (Bourcier et al., 2003; Lopez et al., 2018). The activation of the SDF-1/CXCR4 axis controls cell cycle and regulates apoptosis via the effector molecules, such as NFκB (Helbig et al., 2003; Bamdad et al., 2017). SF further transform into highly contractile myofibroblasts, which express fibronectin receptors (α5β1 and αvβ3 integrins), that promote the assembly of fibronectin fibrils to conduct mechanical force during wound matrix contraction (Jester et al., 1999). Myofibroblasts have altered KSPG synthesis compared with keratocytes, and produce excessive and abnormal ECM components (for example, collagen I, fibronectin, and biglycan) that are deposited in a disorganized manner. Overproduction of abnormal ECM compromises corneal transparency. In addition, myofibroblasts can be generated from bone marrow-derived fibrocytes (de Oliveira and Wilson, 2020). These cells may retain steroid sensitivity, giving a lineage of steroid-responsive myofibroblasts, whereas steroid-resistant cells could be derived from SF or keratocyte-related precursors (Wilson, 2012). However, this hypothesis needs to be validated with more experiments.

Fibrosis occurs when myofibroblasts appear and produce excessive and disorganized ECM, which interferes or blocks the light passage. Transient haze from corneal abrasion or ultraviolet (UV)-mediated riboflavin crosslinking can be reversed by the regeneration of damaged epithelium and basement membrane, which usually occurs within days (Santhanam et al., 2017; Wilson, 2019). The healed epithelium reduces the supply of secreted TGFβ and PDGF, limiting the viability of myofibroblasts and their precursors, ultimately triggering apoptosis before they accumulate inside the stroma and deposit disorganized ECM (Wilson et al., 2007). In a rabbit model of photorefractive keratectomy (PRK), restoring basement membrane structure and function generated spotty areas of clear “lacunae” within the fibrotic opacity, indicating the withdrawal of myofibroblasts (Marino et al., 2017). Subsequent repopulation of keratocytes then induced the paracrine IL-1α-mediated apoptosis of myofibroblasts and remodeling of disorganized ECM to restore the transparency (Kaur et al., 2009). The resolution of corneal fibrosis can occur over months to years and produce either partial or complete recovery of corneal transparency (Torricelli et al., 2016; Wilson et al., 2017). If the epithelium and basement membrane do not regenerate quickly, stromal metabolism becomes dysregulated, leading to keratolysis, collagen fibril disorganization, and eventually corneal fibrosis and scarring. The continuous presence of pro-fibrotic cytokines from prolonged and severe inflammation or infection will promote the differentiation of myofibroblasts, resulting in chronic scarring. If this occurs in the visual axis, vision will be impaired. In this case, corneal tissue replacement becomes necessary to restore vision (Deshmukh et al., 2020).

Pathological conditions in the cornea also affect innervation. Corneal nerves produce neurotrophic factors such as substance P, calcitonin gene-related peptide, epidermal growth factor, nerve growth factor, brain-derived neurotrophic factor, and neurotrophins (Shaheen et al., 2014), that maintain normal cornea and contribute to corneal wound healing (neurotrophic functions). Corneal damages affecting the supply of supportive neuropeptides or directly disrupting the neural circuit lead to neurotrophic keratopathy. This corneal nerve impairment results in epithelial defects, stromal ulceration, and scarring (neurotrophic keratitis) (Shaheen et al., 2014). Abnormal nerve regeneration often predisposes patients to pain, dry eye, and vision loss. This has been demonstrated by the interplay between corneal nerves and immune cells (Sarkar et al., 2013; Seyed-Razavi et al., 2014), between nerves and corneal epithelium (Beuerman and Schimmelpfennig, 1980), and the interaction between nerves and keratocytes, fibroblasts or myofibroblasts. In several reports, human SF (but not keratocytes) promoted neurite outgrowth in a cultured chick dorsal root ganglion cell model, while myofibroblasts delayed neurite growth (Jeon et al., 2018; Yam et al., 2017). The elongating neurites made contacts with myofibroblasts (but not SF), mediated by the phosphorylation of collapsin response mediating protein 2 caused by myofibroblast-secreted TGFβ1 (Jeon et al., 2018). On the other hand, mitomycin C (MMC) treatment after PRK decreased myofibroblast differentiation and resulted in rapid neuronal regeneration (Jeon et al., 2018). Hence, blocking myofibroblast differentiation during early wound healing is critical for restoring corneal innervation to the epithelium and stroma. Further studies identifying molecules involved in this regulation by SF versus myofibroblasts and their associated glia (non-myelinating Schwann cells) could provide new insights into stromal nerve regeneration.

## Topical Corticosteroids

Topical corticosteroids, such as prednisone acetate ophthalmic eye drops, minimize fibrosis through its downregulating TGFβ-mediated inflammatory and fibrotic events (Wen et al., 2002). The scar tissue deposition after PRK was reduced by steroid treatment (Tuft et al., 1993). In injured corneal tissues, the application of corticosteroids prevented macrophage and lymphocyte accumulation, and reduced collagen deposition by fibroblasts (Wangoo et al., 1997). Steroids also act as an immune-suppressant to block the release of inflammatory mediators and inhibit cytokine production (Nur Akkaya and Erbas, 2019). However, the use of steroids has been controversial in active cases of corneal fibrosis associated with bacterial infection. Early treatment can result in uncontrolled infection and worsening of the disease (Herretes et al., 2014). Hence, the infection must be adequately controlled prior to steroid treatment. Frequent instances of abuse and misuse also cause serious local and systemic side effects. Absorption of steroids may increase the risk of developing steroid-induced glaucoma, cataract, and delayed wound healing (Weijtens et al., 2002). Hence, it is necessary to evaluate the patients carefully before steroid treatment.

## Topical Mitomycin C (MMC)

MMC is a classical DNA damaging agent, that forms covalent linkage with DNA, inhibiting DNA replication, transcription, and protein synthesis, causing irreversible senescence of cells (McKenna et al., 2012). For years, it has been used as an anti-cancer drug. In ophthalmology, topical MMC is widely used intraoperatively or shortly after injury to inhibit TGFβ and PDGF-driven proliferation of myofibroblasts and precursors, resulting in apoptosis and retarding the development of fibrosis, such as preventing post-ablation haze after PRK (Teus et al., 2009; Kwok et al., 2019). In addition, MMC is used to prevent the recurrence of pterygium after excision surgery and to treat ocular surface neoplasia (Prabhasawat et al., 2005; Young et al., 2013; Chan et al., 2015). It is applied before, during, or after the excision of neoplasia or pterygium. The intraoperative use is usually in the form of MMC solution soaked in a sponge whereas the subconjunctival injections or eye drops can be performed postoperatively. It is noteworthy that the increased MMC exposure to corneas has a higher risk of complications, such as delayed epithelialization, scleral calcification, ulceration, necrotizing scleritis, and damage to the corneal endothelium and ciliary body (Nassiri et al., 2008; Kam et al., 2015).

## SMAD Signaling Blockers

TGFβ isoforms (β1 and β2) regulate fibrosis and scarring through the differential activation of canonical TGFβ/Smad signaling (Walton et al., 2017). Ligand binding activates TGFβ receptors through dimerization and trans-autophosphorylation, after which the cytoplasmic effectors are phosphorylated. These include Smad2/3 proteins, which translocate to the nucleus and activate gene transcription associated with fibrosis and cell metabolism. Rosiglitazone, a small molecule agonist of peroxisome proliferator-activated receptor gamma (PPARγ), blocks the nuclear translocation of Smad2, reducing TGFβ-mediated fibrosis and myofibroblast development (Burgess et al., 2005; Saika et al., 2007). In a feline PRK study, topical rosiglitazone reduced the backscattering of anterior stroma and corneal reflectivity as assessed by optical coherent tomography and decreased myofibroblast density (detected by reduced expression of α-smooth muscle actin, αSMA), without affecting corneal re-epithelialization or stromal recovery (Huxlin et al., 2013). However, as rosiglitazone also acts as an insulin sensitizer for type 2 diabetes mellitus treatment, there could be risks of systemic effects, such as congestive heart failure and bladder cancer (Komajda et al., 2010). On the other hand, platelet-rich plasma (PRP) extracted from autologous blood has been reported to enhance corneal wound healing and stimulate epithelial regeneration (Tanidir et al., 2010; Etxebarria et al., 2017). PRP contains high concentrations of essential growth factors and cell adhesion molecules that contribute to tissue healing. Different studies have shown that topical PRP successfully treated dormant ulcers (hard-to-heal epithelial defects), dry eye syndromes, and ocular surface syndrome post Laser In Situ Keratomileusis (LASIK), as well as promoted surface reconstruction after corneal perforation associated with amnion transplantation (Alio et al., 2012; Alio et al., 2017; Rechichi et al., 2020). However, for stromal wound healing in a rat model, PRP treatment suppressed stromal cell apoptosis and increased myofibroblast generation by TGFβ/SMAD3 activation (Koulikovska et al., 2015). Hence, PRP seems to be effective in enhancing epithelial wound healing and promoting ocular surface regeneration in different pathological conditions. However, it has a controversial ability on stromal scar management. Though PRP preparation with low content of leukocytes and red blood cells can be standardized following the guidelines of good clinical practice, the biological contents, such as growth factors, vary widely among different sources (Gomez et al., 2015). This limitation warrants further exploration to optimize and quantify the beneficial PRP-derived cytokines and growth factors for their potential application in regenerative medicine.

## Vitamin C

Vitamin C (sodium ascorbate) has been known to facilitate collagen synthesis and deposition by keratocytes and to produce parallel arrays of ECM fibrils (Grobe and Reichl, 2013). Its anti-oxidant property reduces corneal neovascularization (Lee and Chung, 2012). In a clinical trial of patients with infectious keratitis, intravenous vitamin C treatment improved corneal epithelial wound healing and reduced haze in terms of density and size (Cho et al., 2014). However, differing outcomes were achieved in patients with laser-induced corneal injury. While Stojanovic et al. (2003) observed a prophylactic effect against haze development after PRK by oral ascorbic acid, Yulish et al. (2012) reported a null effect, in addition to the perioperative MMC (Stojanovic et al., 2003; Yulish et al., 2012). This suggests that the treatment effect may depend on the disease etiology and the underlying mechanisms of how ascorbate modifies corneal haze in different conditions need to be further investigated. Although there is a lack of randomized clinical trials to support the use of ascorbate in clinical practice, oral vitamin C is prescribed to patients after PRK and ocular chemical injury to promote epithelial wound healing.

## Recombinant Proteins and Inhibitors

The cornea is an ideal tissue for gene and protein therapies, as it is easily accessible by topical application or injection via subconjunctival, intracameral, or intrastromal routes. Regarding particular mechanisms of action, protein-based pharmaceuticals can address corneal surface integrity, tear composition, wound healing, inflammation, haze and scarring, nerve regeneration, and neovascularization. The topical administration of drug usually has a lower risk of systemic side effects due to the limited absorption into the bloodstream, as opposed to intravenous or intramuscular injections. In a rabbit keratectomy model, topical application of recombinant human bone morphogenetic protein (BMP)-7 suppressed TGFβ-related fibrosis, as observed by the reduced differentiation of αSMA-positive myofibroblasts (Chung et al., 2017). As a member of TGFβ superfamily, BMP7 mediates Smad-1/5/8 signaling, suppressing Smad2 phosphorylation to counteract the fibrotic effect of TGFβ/Smad signaling. Other studies have also reported that BMP7 upregulation via DNA-coated gold nanoparticles or adenoviral vectors inhibited corneal fibrosis and suppressed myofibroblast proliferation after alkali injury on corneas (Saika et al., 2005; Tandon et al., 2013).

In a murine model of microbial keratitis, topical decorin reduced corneal opacities, with the downregulation of αSMA, fibronectin, and laminin, and the treatment promoted the healing of damaged epithelium (Hill et al., 2018). Decorin is a small leucine-rich proteoglycan that is naturally present inside the corneal stroma. This molecule binds with high affinity to collagen fibrils and regulates the fibrillar spacing in maintaining corneal transparency (Kalamajski and Oldberg, 2010; Mohan et al., 2011). Moreover, decorin binds TGFβ and sequesters it in the ECM, potentially inhibiting the pro-fibrotic TGFβ activity. Decorin also modulates the action of growth factors, including vascular endothelial growth factor (VEGF), and PDGF, hence modulating the initiation and progression of neovascularization and haze (Grisanti et al., 2005). Its regulation of MMPs and tissue inhibitors of metalloproteinase could also trigger fibrolysis, affecting scar formation (Ahmed et al., 2014; Hill et al., 2015).

Immunoglobulin CD147, also known as ECM-MMP inducer (EMMPRIN) or basigin, promotes TGFβ-mediated myofibroblast differentiation (Huet et al., 2011). This molecule is predominantly expressed in the corneal epithelium but is markedly induced in the anterior stroma of ulcerated corneas, and induced MMP expression (including MMP-1, 2, and 9) at the epithelio-stromal boundary (Gabison et al., 2005). Sustained upregulation of EMMPRIN and MMPs could lead to excessive matrix degradation, delayed wound healing, and may even result in corneal melts. Topical administration of EMMPRIN inhibitor, SP-8356, suppressed the myofibroblast population and the deposition of abnormal ECM products (e.g. MMP9, Col3, and Col5) in a rat model of corneal alkali injury, hence reducing corneal haze and fibrosis (Joung et al., 2020). Moreover, green tea polyphenol epigallocatechin-3-gallate (EGCG), was reported to restrain EMMPRIN and MMP-9 expression via 67-kD laminin receptor pathway in a PMA-induced macrophage system (Wang et al., 2016). From different studies, EGCG has a profound safety profile and bioavailability in cells and animal tissues and is known to suppress a variety of inflammatory and angiogenic factors, including NF-κB, IL-1β, COX2, VEGF, and MMPs (Lee et al., 2011; Sanchez-Huerta et al., 2011; Miyagawa et al., 2020). Therefore, the administration of EGCG to corneal wounds may represent a therapeutic alternative to suppress corneal inflammation and fibrosis. In a mouse model of acute alkali burn, topical application of EGCG to the damaged corneas significantly reduced inflammation, edema, opacities, and neovascularization while promoting corneal epithelial healing (Gulias-Canizo et al., 2019).

Blocking myofibroblast proliferation could be a potential therapeutic strategy to prevent corneal fibrosis. Intermediate-conductance calmodulin/calcium-activated potassium channel 3.1 (KCa3.1) regulates cell cycle progression and proliferation. Its expression has been reported in the development of fibrosis of the cornea and different organs (Huang et al., 2013; Roach et al., 2015). Treatment with the KCa3.1 inhibitor, TRAM-34, reduced the expression of pro-fibrotic markers, such as αSMA, in the corneal stroma (Anumanthan et al., 2018).

## Regenerative Cytokines

**FIGURE 1:**
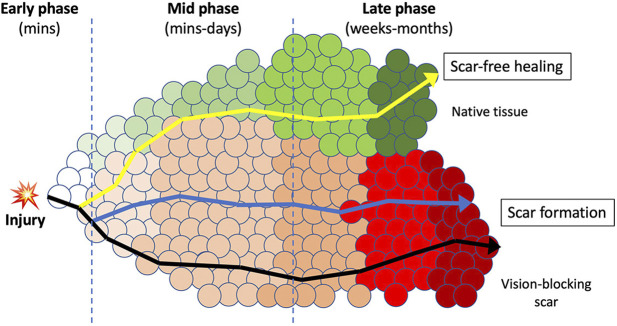
A schematic diagram illustrating the early intervention therapy to re-route the wound healing and scarring response. A continuous spectrum of micro-niches (circles) that represent molecular and phenotypic events that constantly changes over time after injury. The route passes through these processes and the final phenotypic outcome is dependent upon the micro-niches in which resident and infiltrating cells are interacting with the extracellular regions. In adult tissue, upon injury, the normal transition through these sequential micro-niches usually ends up in scar formation (dark line). In corneas, this will block the light passage and results in vision loss. Medications modulating minor pathways could alter the micro-niches, but still results in similar phenotypic outcome of scar formation (blue line). Intervention at early time after wounding could alter the wound microenvironment and produce a “re-routing” through a different series of processes that could result in scar improvement or scar-free healing, and regeneration of native tissues (yellow line). This can improve the corneal clarity and restore vision.

Early re-routing of the scar-forming process could be beneficial for scar-free healing and should occur a short time after injury, ideally before the scarring process initiates. The process of wound healing can be described as changes of the microenvironment that involve a multitude of dynamic and interactive molecular and phenotypic events initiated after injury (Figure 1). This process represents a continuous and overlapping spectrum of interactions between cells (including resident cells and infiltrating cells), and extracellular regions that constantly change over time (collectively termed as micro-niches, represented by circles in Figure 1). After injury, pathways preferentially spanning through these micro-niches result in scar formation. Even though conventional injury management could alter the routes, the tissue still develops a similar final scarring phenotype. If early intervention is given soon after injury (e.g. to inhibit inflammation and fibrosis), it has the potential to “re-direct” the healing response towards a scar-reducing or scar-free phenotype. This strategy can be made possible by establishing high levels of anti-scarring cytokines (or therapeutic proteins) relative to the levels of pro-scarring molecules. In different tissues (including the cornea), members of the TGFβ family can activate or inhibit fibrosis, mechanistically acting through both canonical TGFβ/Smad and non-Smad pathways. Among them, TGFβ1 and β2 isoforms promote fibrosis and scarring, whereas TGFβ3 inhibits scarring and drives scar-free healing effects (Penn et al., 2012; Gilbert et al., 2016). Our group has reported that human CSSC produce TGFβ3 when they were applied to acute corneal wounds, hence restoring clear cornea tissues (Weng et al., 2020). CSSC with TGFβ3 knockdown by small interfering RNA method lost this scar-reducing effect. Other studies have also shown that TGFβ3 stimulates the non-fibrotic stromal matrix production in corneas and maintained consistent collagen fibril organization and spacing (Karamichos et al., 2011; Karamichos et al., 2013). Scarless healing is well documented in amphibians, which can regenerate tissues and even limbs following injury or amputation. In fact, skin wounds in mammalian embryos at the early gestation stage can heal without scarring and regenerate native dermal matrix, while scar-forming healing happens in late gestation and afterward (Satish and Kathju, 2010). Such scarless healing is associated with an increased expression level of anti-fibrotic TGFβ3 relative to the pro-fibrotic TGFβ1 (Eslami et al., 2009). While the ratio is high in fetal tissues that heal without scarring, it is lower in tissues with scar-forming healing. A higher TGFβ3/β1 ratio is also found in postnatal oral mucosa, which heals rapidly and without scarring (Schrementi et al., 2008). Hence, methods to increase TGFβ3/β1 ratio soon after injury have merit to control the onset of scarring and could re-direct tissue healing towards a scar-reducing or scarless pathway. After injury, the activation of latent pro-fibrotic TGFβ occurs at different time points: immediately after wounding and during re-epithelialization. Infiltrating cytokines from the damaged corneal epithelium and Bowman’s layer are the primary sources of active TGFβ immediately after injury. These molecules act as potent chemo-attractants for inflammatory cells and macrophages to invade into the wound site, further activating the latent TGFβ during re-epithelialization, which usually occurs within hours to a few days. Hence, the immediate phase after injury represents a possibly unique opportunity to control pro-fibrotic TGFβ, and this could influence the healing outcome. If their bioavailability can be suppressed early enough, the pathways leading to tissue inflammation and fibrosis could be redirected to a less-fibrotic or even a non-fibrotic repair (Figure 1).

Pro-fibrotic TGFβ informs could be controlled by the use of synthetic inhibitors that interfere with TGFβ bioavailability, ligand/receptor interaction, and/or receptor kinase activity (Huynh et al., 2019). However, these interventions could broadly affect the activity of all ligands that act through the transmembrane TβRI and TβRII receptors, including TGFβ3. Hence, a need exists for an orthogonal means of downregulating pro-fibrotic TGFβ. One option is the soluble amnion membrane extract (AME). *In vitro* stromal keratocyte culture studies have shown that the human amnion matrix and its soluble protein extract reduced TGFβ1 and β2-related signaling (Tseng et al., 1999; Espana et al., 2003; Kawakita et al., 2005; Yam et al., 2015; Fenner et al., 2019). Cultured keratocytes had a minimal transition to fibroblastic phenotypes during early passages, even in the presence of serum. AME also suppresses cell apoptosis, angiogenesis, and microbial growth, and it promotes immune responses that are advantageous for tissue regeneration (Dua et al., 2004; Shay et al., 2011). Results of a clinical trial using AME eye drops (NCT02746848) have shown its beneficial outcomes in promoting corneal wound healing, pain relief, and anti-inflammatory activity (Murri et al., 2018).

Moreover, hepatocyte growth factor (HGF) is an anti-fibrotic molecule that reduces fibrosis in various organs (Okayama et al., 2012; Miyagi et al., 2018). In a murine lung fibrosis model, its administration triggered myofibroblasts to undergo apoptosis by degrading the ECM-myofibroblast anchors through MMP activation (Mizuno et al., 2005). HGF activates Smad7 to prevent Smad2 phosphorylation, hence inhibiting pro-fibrotic TGFβ signaling. In the eye, HGF is expressed in corneal epithelium, stroma, and endothelium, as well as the lacrimal gland. Mittal et al. (2016) have reported that administration of exogenous HGF restored corneal transparency in a murine corneal wound model. Whether HGF could reduce or even reverse corneal scarring needs to be investigated.

## Anti-Fibrotic microRNAs and CSSC Exosomes

MicroRNA therapeutics is becoming an important field in medical research, due to the pleiotropic nature of microRNAs, which make them particularly attractive as candidate targets for diseases with a multifactorial origin and no effective treatment. A growing number of reports support the use of miRNAs as biomarkers for diagnosis, and drug intervention (Recchioni et al., 2013; Scott et al., 2015; Sun et al., 2015; Reid et al., 2016; Montastier et al., 2019). MicroRNAs (miRs) are a class of small non-coding RNA with about 21-22 nucleotides in length. They regulate target gene expression by complementary binding to the 5’ or 3’ untranslated region (UTR) of target mRNAs. In cases of high to complete base-pairing between miRNAs and target mRNAs, argonaute-2 in the RNA-induced silencing complex cleaves the target mRNA strands, resulting in gene silencing. When complementarity is lower, miRNA binding can suppress the translation efficiency to downregulate target gene expression. Several reports have described miRNA changes in corneal disorders. The expression of miR-133b was correlated with pro-fibrotic genes in rabbit corneas following excimer laser ablation (Robinson et al., 2013). Signature miRNAs have been identified to associate with corneal neovascularization (Mukwaya et al., 2019). While many articles have extensively reviewed miRNAs in fibrosis of different tissues and summarized their potential regulation of signaling pathways that regulate fibrosis events (Bardin et al., 2018; Jiang et al., 2010; O'Reilly, 2016; Patel and Noureddine, 2012; Vettori et al., 2012), we pay special attention to miRNAs in corneal stromal fibrosis. In a recent study using murine wound corneas, extracellular vesicles (EV) or exosomes derived from human CSSC showed a therapeutic correction of corneal scarring (Shojaati et al., 2019). When CSSC-derived EV were depleted of miRNA content using Alix knockdown to reduce ESCRT (endosomal sorting complexes required for transport) pathway in exosome maturation, they were ineffective to block corneal scarring and lost their regenerative function. It is possible that miRNAs inside EV could affect the paracrine function (Sun et al., 2018). Precursor miRNA (pre-miRNA)-bound processing complex (such as Dicer, argonaute, and TRBP) are found inside EV, where they continue to generate mature miRNAs (Tran, 2016). This could establish the source of miRNAs inside EV. Hence, CSSC could produce and deliver miRNAs with the anti-fibrotic capability to regenerate transparent corneal tissue after injury. Using Nanostring analysis, Funderburgh et al. have identified a list of miRNAs present in CSSC-derived EV (Table 1) and filed in WIPO (PCT) WO2019169380A1 (Stem cell-derived exosomes for treatment of corneal scarring. 2019. Inventors: Deng SX and Funderburgh JL) (https://patentscope.wipo.int/search/en/detail.jsf?docId=WO2019169380).

**TABLE 1:** Lists of microRNAs present in exosomes derived from human corneal stromal cells that function to reduce corneal scarring.

Reported microRNAs in tissue fibrosis and inflammatory responses	Novel microRNAs
Let-7b, -7l, miR-100, 103/107, 1246, 125b, 1290, 1297, 1306, 133a, 134, 138, 141, 143, 145, 146a, 146b, 155, 16, 196a, 199a, 200c, 208a, 21, 212, 221/222, 224, 27b, 29, 320e, 323a, 363, 370, 378, 379, 381, 411, 4492, 485, 495, 498, 520c, 532, 543, 590, 598, 630, 654, 665, 761, 891, 92a, 92b	miR-1197, 125a, 1257, 1261, 1286, 1295a, 151a, 191, 206, 25, 376a, 409, 423, 4301, 431, 4455, 4532, 493, 502, 514b, 539, 548ah, 548g, 549a, 556, 574, 585, 587, 612, 613, 626, 644a, 6721, 769, 888, 889

We anticipate that not every miRNA identified in CSSC-EV will influence fibrosis and scar reduction. Some miRNAs can be redundant, while miRNAs with relevant activity may work in groups or combinations. This makes the identification of specific miRNAs difficult and complicated, as well as costly in setting up experiments for validation. Bioinformatics approaches, such as target gene search and enriched pathway prediction, should be employed to identify single or groups of miRNAs that regulate inflammation, fibrosis, and/or immune responses to subsequently validate using *in vitro* and *in vivo* models. Target gene search for each miRNA is available using online databases, such as TargetScan (http://www.targetscan.org/vert_72/) and miRDB (http://mirdb.org/). Since each platform uses proprietary algorithms in calculating the binding score to a target sequence, the use of more than 1 platform can increase the confidence in hits identified independently while compensating for the limitations of each approach. The top list of genes shared in the output from both platforms can then be selected for enriched pathway analyses. Since one or several miRNAs could mediate gene regulatory effects, the gene lists from different miRNA candidates should be combined and imported for enriched gene ontology terms and pathway analysis using tools, such as DAVID (Database for Annotation, Visualization and Integration Discovery) (https://david.ncifcrf.gov/), KEGG (Kyoto Encyclopedia of Genes and Genomes) (https://www.genome.jp/kegg/pathway.html) and IMPALA (Integrated Molecular Pathway Level Analysis) (http://impala.molgen.mpg.de/). After the removal of non-coding genes, the remaining gene candidates will be examined for over-representation in specific signaling pathways, biological processes, and phenotypes.

Due to the vast number of miRNAs upregulated in CSSC EV that reduce corneal scarring (Table 1 and filed in WIPO (PCT) WO2019169380A1), we select several candidates to discuss their potential roles in tissue fibrosis.

### miR-29

The expression level of miR-29 in tissue is low during embryonic stages but increases with age, reaching a high level in the mature tissues, such as liver, kidney, lung, and heart (Cushing et al., 2011; Kamran et al., 2015). Multiple factors can regulate miR-29 expression, at both transcriptional and post-translational levels. NFκB transcription factor YY1 and c-Myc directly bind to miR-29b2/c promoter, inhibiting miR-29 expression (Mott et al., 2010; Wang et al., 2008). Various pro-fibrotic cytokines also regulate miR-29 expression (Cushing et al., 2011; Maurer et al., 2010; Ogawa et al., 2010). In lung and cardiac fibroblasts, TGFβ and PDGF downregulated miR-29, possibly mediated via Smad3 pathway (Divakaran et al., 2009; Maurer et al., 2010). Hence, miR-29 is considered as a “master fibro-miRNA” regulator with organ specificity, and reduced miR-29 expression has been found in the fibrosis of multiple organs (Bian et al., 2014; Deng et al., 2017). The human miR-29 family has 3 members, miR-29a, b, and c, encoded by 2 gene clusters (chr. 7q32.3 and chr. 1q32.2 in humans). They share common seed sequences and are predicted to target largely overlapping sets of genes. However, they exhibit differential regulation, depending on their subcellular distribution. miR-29a is primarily localized in the cytoplasm with some nuclear presence. In contrast, miR-29b is significantly enriched inside the nucleus (Hwang et al., 2007). It remains to be shown whether regional differences of miRNA expression level are related to their interaction with different proteins and whether those differences elicit biological effects. At least 16 ECM genes are the direct targets of miR-29, providing a dramatic example of a single miRNA targeting a large group of functionally related genes. They code for key proteins involved in the physiological or pathological formation of ECM, including a number of collagen isoforms, laminin γ1, fibrilin 1, elastin, MMP-2, and integrin β1 (Kwon et al., 2019; Liu et al., 2010; van Rooij et al., 2008). Knockdown of miR-29 in canine atrial fibroblasts significantly upregulated Col1A1, 3A1 and fibronectin (Dawson et al., 2013). However, the overexpression or knockdown of miR-29 did not alter αSMA expression or cell number of myofibroblasts (Qin et al., 2011). Thus, the miR-29 family negatively regulates fibrosis by targeting collagen matrix synthesis rather than by inhibiting myofibroblast. Table 2 summarizes miR-29 expression in different fibrotic diseases. However, limited studies about miR-29 regulation were conducted in corneas. Downregulated miR-29 level was detected in clinical Fuchs’ corneal endothelial dystrophy (FECD) specimens while a wide range of miR-29 target genes was upregulated, suggesting a role of miR-29-related deposition of ECM in the pathogenesis of FECD (Matthaei et al., 2020). Overexpression of miR-29b suppressed ECM-related Col1A1, Col4A1, and laminin C1 expression in human corneal endothelial cells (Toyono et al., 2016).

**TABLE 2:** miR-29 expression in fibrotic diseases.

Tissues/diseases	Reported functions
Cardiac fibrosis	Reduced ECM fibrosis genes (COL1, COL3, FN1, elastin, SMAD2/3) Zhou et al. (2012)
	Decreased expression in cardiac hypertrophy Li et al. (2016), myocardial infarction van Rooij et al. (2008)
	Induced myocardial cell apoptosis (target gene: Mcl-2) Ye et al. (2010)
Pulmonary fibrosis	Negative association to fibrosis severity (COL3A1, COL4A1) and control TGFβ1-independent fibrosis gene expression (ADAMS, laminins, integrins) Cushing et al. (2011)
Hepatic fibrosis	Suppressed COL1, and ECM maturation Ogawa et al. (2010)
	Prevented stellate cell activation in liver fibrosis Matsumoto et al. (2016)
	Inhibited PDGF-C, IGF1 expression as an anti-fibrogenic mediator Kwiecinski et al. (2012)
Renal fibrosis	Suppressed TGFβ/Smad3 signalling (COL2A1, HIF1a, Spry1, TPM1a downregulation) Qin et al. (2011) miR-29c in urine exosomes as a predictor of early renal fibrosis in lupus nephritis Sole et al. (2015)
	miR-29b attenuated histone deacetylase 4-mediated podocyte dysfunction and renal fibrosis in diabetic nephropathy Gondaliya et al. (2020)
Systemic sclerosis	Targeting TGFβ-activated kinase 1 binding protein 1 to reduce TIMP1 expression in dermal fibroblasts Ciechomska et al. (2014)
	Induced apoptosis of dermal fibrosis via Bax:Bcl2 ratio Jafarinejad-Farsangi et al. (2015)
	Reduced miR-29a in bleomycin model of skin fibrosis Maurer et al. (2010)
Keloids	Prevented collagen accumulation in skin fibroblasts Zhang et al. (2016) miR-29 mimic repressed skin fibroplasia Gallant-Behm et al. (2019)

### miR-107

It belongs to a rare class of miRNAs that are proposed to regulate other miRNAs. In a study of multiple miRNA interactions, miR-107 was found to directly interact with let-7 and form an internal loop of let-7/miR-107 duplex, stabilizing let-7 activity and facilitating its target downregulation. The let-7 family has been shown to regulate IL-13 in cultured T-cells and a murine model of allergic airway inflammation. Suppressing let-7 family-induced inflammatory responses (Kumar et al., 2011). In corneas and dry eye disorders, keratoconjunctivitis was associated with up-regulated IL levels, including IL-13 (Pflugfelder et al., 2013; Imai et al., 2017), which promotes fibrosis (Doherty and Broide, 2007). We hypothesize that the inflammation- and fibrosis-related IL-13 expression can be regulated by let-7, which is stabilized by miR-107. Moreover, miR-107 was downregulated in response to pro-inflammatory LPS treatment, probably due to toll-like receptor (TLR) signaling in macrophages, leading to increased cell adhesion via CDK6 (Hennessy et al., 2011). In corneas, miR-107 negatively regulated p38/AP-1 pathway via its targeting on mitogen-activated protein kinase 7 and promoted the differentiation of limbal epithelial cells (Park et al., 2015).

### miR-155

Following the exposure of immune cells to inflammatory cues, signals are sent to cell nuclei resulting in dynamic transcriptional changes of immune responsive genes, which constitutes the first step of coordinated inflammatory responses. Besides the altered protein expression, there are changes in miRNA-producing transcripts (O'Connell et al., 2012). miR-155 is identified as an inflammatory miRNA that is upregulated by NFκB through TLR signaling (O'Connell et al., 2007; Kurowska-Stolarska et al., 2011). Its genomic location is mapped within a region known as the B-cell integration cluster on chr. 21 (Faraoni et al., 2009). The expression of miR-155 regulates the inflammatory mediators in macrophages to promote plaque formation and rupture and downregulates the negative regulators SHIP1 and SOCS1, thus activating AKT and interferon (IFN) response genes (Androulidaki et al., 2009). MiR-155 also enhanced the expression of p-STAT and PDCD4, and IL-6 and TNFα production. These findings suggest that miR-155 might mediate inflammation via the SOCS1-STAT3-PDCD4 axis. Mice with miR-155 knockout had a defect in inflammatory T-cell development, possibly mediated by cMaf expression (O'Connell et al., 2010). Higher expression of cMaf after miR-155 knockout could hinder the development of Th1 or Th17 cell types, regulating the inflammatory response (Rodriguez et al., 2007). In silico analysis also showed that PU.1, a miR-155 target gene, was important for the early activation, maturation, and regulation of B cells in hematopoiesis. Hence, a strategy to modify intracellular miR-155 levels could have a therapeutic potential to regulate inflammation and immune responses. In corneas of fungal keratitis, the dysregulation of miR-155-5p has suggested the involvement of TLR pathway in tissue healing (Boomiraj et al., 2015).

### miR-381

As the first line of defense, innate immunity plays a key role in preventing invasion by pathogens. TLRs can directly sense the foreign invaders through microbial components (e.g. lipopolysaccharides) and trigger an innate immune response (Kesar and Odin, 2014). TLR signaling activates NF-𝜅B, which upregulates inflammatory cytokines. NF-𝜅B activation requires the phosphorylation and degradation of inhibitor 𝜅B (I𝜅B) proteins, including two kinases, I𝜅Bα and I𝜅Bα. I𝜅Bα can bind the NF𝜅B p65 subunit, which is prevented from nuclear translocation and induction of pro-inflammatory cytokine expression. MiR-381 regulates NF𝜅B signaling by targeting I𝜅Bα, thereby regulating pro-inflammatory TNFα, IL-6 and COX-2 (Xu et al., 2015). Blocking the NF𝜅B pathway has been shown to suppress corneal inflammation and angiogenesis (Lennikov et al., 2018). However, the expression of miR-381 in corneal tissue has not been reported.

### miR-543

This miRNA clusters on chr. 14q and targets the epigenetic regulator Tet1 and Tet2, which play a direct role in DNA methylation and gene activation. It also has an indirect role in protein acetylation. TET enzymes convert 5-methylcytosine (5hmC) to 5-hydroxymethylcytosine in DNA. *Tet1* and *Tet2* are Oct4-regulated enzymes that sustain 5hmC in mouse embryonic stem cells (ESC) and are induced concomitantly with 5hmC during the reprogramming of fibroblasts to induced pluripotent stem cells. ESC depleted of *Tet1* by RNAi treatment had reduced expression of Nodal antagonist *Lefty1*, and displayed hyperactive Nodal signaling and skewed differentiation into the endoderm-mesoderm lineage in embryoid bodies *in vitro* (Fuentes-Mattei et al., 2020). Their expression was regulated by Oct4/Sox2 complex, hence in association with stem cell pluripotency, and the depletion of Tet1 impaired the self-renewal and differentiation of ESC (Koh et al., 2011; Williams et al., 2011). The presence of Tet1 and Tet2 maintains the homeostasis of mesenchymal stem cells through demethylation of P2rX7 to control exosome and miRNA release (Yang et al., 2018). Human CSSC express and release miR-543 via exosomes. However, the action of this miRNA on target stromal tissue regeneration or on any other administered CSSC in terms of cell survival, differentiation, and maintenance in host tissue needs to be further investigated.

## Perspectives and Challenges of Developing Therapeutic microRNAs

Many reports have illustrated the changes or dysregulation of miRNAs in different types of disorders, including cancers, tissue injuries, and age-related diseases (Iorio and Croce, 2012; Nies et al., 2021; Pinel et al., 2019). There has been growing interest in developing miRNA therapeutics via miRNA binding and regulating target gene expression, which modulates the disease-associated cellular events. Currently, the therapeutic uses of small molecule inhibitors or monoclonal antibodies have shown promise in manipulating specific gene expression (Jamilloux et al., 2019; Zarrin et al., 2021). However, there are many genes that are not “druggable” with these methods. Therapy with miRNAs offers the ability to target different genes in a given pathway. For example, miR-29 targets a broad spectrum of ECM and pro-fibrotic molecules, including multiple types of collagen, PDGF, thrombospondin, and SPARC, suggesting that miR-29 could broadly regulate tissue fibrosis and ECM remodeling (Cushing et al., 2011). In regulating target gene expression, the use of specific antagomirs (or anti-miRs), which are chemically synthesized oligonucleotides with complementary binding to a specific miRNA, achieves miRNA silencing. On the other hand, miRNA mimics increase the expression level of a particular miRNA, downregulating the expression of target genes, such as oncogenes in carcinogenesis.

Though miRNAs have features that can be developed for drug design and efficacy, there are challenges that need to be overcome before considering this strategy for clinical uses. A major obstacle is that miRNAs exhibit pleiotropy in multiple senses (one miRNA can regulate multiple genes and a given gene can be regulated by more than one miRNA), making their therapeutic use potentially very complex (Yang et al., 2018). Complex miRNA targeting could in turn affect the regulation of different mRNA molecules. Hence, more defined information on miRNA-involved cellular processes, including a clear picture of regulatory networks of miRNAs and target genes, is required. The short length of miRNAs makes them highly prone to degradation by nucleases upon their addition to the biological systems (O'Brien et al., 2018). The rapid clearance of naked RNAs leads to a short life and instability. Chemically engineered miRNA modifications would be necessary to protect miRNAs from degradation and promote long-lasting potency (Segal et al., 2020). On the other hand, potential off-target effects of miRNAs (due to the imperfect complementarity with the 3’ UTR of target genes) might cause undesirable silencing of other genes, potential toxicity, and reduced therapeutic efficacy. In addition, double-stranded RNAs (miRNA duplexes) can be recognized as pathogens by the host immune system, triggering inflammatory cytokine release and IFN pathways (Yu et al., 2018). The choice of a suitable delivery vehicle for miRNA should ideally inhibit immune response, improve targeting to the correct tissues and allow control over dosing. Different studies have described the administration of miRNAs in conjugation with modified cationic nanoparticles to enhance tissue penetrance, and escape from endosomal entrapping and degradation by lysosomes (Lee et al., 2019; Salah et al., 2019). In corneas, aside from nanoparticles, miRNAs can be packaged and delivered via exosomes as CSSC-derived EV have been shown to inhibit or reverse corneal scarring (Shojaati et al., 2019). This represents a valuable alternative to tissue- or cell-based therapies due to the minimal risk of rejection. Eye drop formulation and frequency can be adjusted according to the patient’s needs, including the injury type and recovery status.

## Summary

The current standard for treating severe and irreversible corneal scarring in the visual axis often entails a corneal transplant, which carries risks of rejection, infection, and graft failure. Complications also include prolonged rehabilitation, lifelong use of immunosuppressants, and loss of corneal strength, with a considerable human and financial cost. In addition, the worldwide shortage of donor materials has greatly hampered the applicability of corneal transplantations, in particular in under-developed and developing countries. If the density of corneal opacities can be reduced, then other non-surgical therapies (such as the use of contact lenses) can correct corneal astigmatism and provide visual rehabilitation. This will lessen the demand for transplantable donor grafts, which can be reserved for treating serious and dense scars. Early intervention would thus be a valuable approach to delay visual deterioration, and reduce complications, ideally decreasing the need for corneal transplantation. A desirable approach to delay scarring would be to stabilize the wound soon after injury and redirect the healing process to reduce inflammation and fibrosis. The use of regenerative cytokines as a first-line treatment would elevate the expression of anti-fibrotic cytokines and suppress cytokines, reducing tissue inflammation and fibrosis, hence potentially stimulating regeneration of clear stroma. If successful, this would also improve the effectiveness of subsequent treatments to recover visual acuity. On the other hand, different microRNAs with anti-fibrotic activity have been identified, however, their use to reverse or reduce tissue fibrosis is still under investigation. Their specific actions and potential side-effects or contraindications need to be clarified before any of these molecules can be developed for miRNA therapeutics. Besides, these miRNAs can be potentially applied for cell product screening. In cell-based therapy, the cell stocks for clinical application must be stringently selected and quality controlled. Only cells that are carefully screened for correct phenotypes and functions can be released for patient uses. If a panel of CSSC-EV-derived miRNAs with anti-inflammatory and anti-fibrotic properties were characterized, they could be used to define the release criteria for therapeutic CSSC. Their detection in the secretome will have minimal influence on the cell manufacturing process and does not require extra cells for the assays. This will assist medical professionals to justify the use of high-quality cell products for patients.
